# College and Hospital Notes

**Published:** 1911-05

**Authors:** 


### COLLEGE AND HOSPITAL NOTES

Lectures and demonstrations will be continued in the medical department of the University of Buffalo to May 13. The final examinations will begin the following week.

There is every probability that the city will purchase the West Farm site at Kensington avenue and Grider street, for a municipal hospital group of buildings. The common council seems agreed that the price of $225,000 is reasonable and both branches have passed the resolution authorising the purchase.

Work on the J. N. Adam Memorial Hospital at Perrysburg, N. Y., in which provision will be made for the care of 125 cases of incipient pulmonary tuberculosis, is well under way and it is expected the hospital will be opened in the fall. The bill authorising the city to issue additional bonds for $50,000, bringing the cost of the hospital up to $250,000, has been passed by the legislature and signed by Governor Dix.

The new branch at the Sloane Maternity Hospital, New York, to be devoted to gynecologic work was completed during March and is now in use. It is the gift of Mr. W. D. Sloane to the College of Physicians and Surgeons of Columbia University and was erected at a cost of $200,000. The building is composed of eight floors with a roof garden. A feature is the operating clinic, which will be used for the instruction of nurses and students. The maintenance of the branch has been provided for by an endowment in perpetuity from the donor of the building.

The German Deaconess's Society will begin in the near future the erection of its new hospital, plans for which have long received careful consideration. There was needed $200,000 to carry out the provisions of the architect's plans and this the committee in charge of finances planned to raise in a two weeks' campaign during the first fourteen days of April. The exact total was not reached but assurances of financial support in the near future opened the way for a completion of the work and building operations will be begun at once.

Commencement exercises at the University of Buffalo will be held May 30, 31 and June 1, 1911. This year Wednesday, May 31, will be devoted to clinics, instead of two days as in former years. The clinics will consist of a series of short talks on special subjects by different specialists. The program opens Tuesday evening, May 30, with the alumni meeting in Alumni Hall, which will be followed by a collation in the college library. On Wednesday evening, at 7 P. M., the alumni dinner will be held with Dr. Charles Cary as its guest. Thursday, June 1, 11 A. M., Commencement exercises will be held at the Teck Theatre. Monday, May 29, afternoon and evening, will be available for class reunions, dinners, and general relaxation, details to be arranged by the various class organisations. The reunion classes are those of '51, '61, '71, '81, '91, '01.

---

Dr. Elmer Ellsworth Brown, of Washington, United States commissioner of education, since 1906, has been appointed chancellor of the New York University to succeed the Reverend Henry Mitchell MacCracken, resigned.

The appointment was announced by the university council, April 24, 1911, after an executive session, at which the report of a subcommittee recommending Dr. Brown was adopted. The subcommittee had given a year to the consideration of a suitable successor to Dr. MacCracken, whose resignation was announced in February, 1910, and considered many prominent educators before deciding unanimously upon Dr. Brown.

Dr. Henry Mitchell MacCracken, who retires as chancellor of New York University, gave many of the best years of his life to the building up of this educational center. He went to the old University of the City of New York in 1884 as professor of philosophy and vice-chancellor (the chancellorship was then an honorary position) and greatly extended the scope of work done there. In his regime the institution's name was changed, the seat was moved to University Heights and the Hall of Fame for Great Americans was established. Dr. MacCracken retires in his seventy-first year.

---

The State Library at Albany which has been all but destroyed by fire was the great instrument of the intellectual and moral culture of the state. Its collections related to every subject and reached out to every moral, professional, commercial and industrial interest of the commonwealth. Its law library was beyond the ordinary: it provided what ordinary law libraries could not furnish. So with its medical, technological, genealogical, theological, educational and other collections. Its books were sent not only to all manner of organisations engaged in culturing study, but freely to individuals in every town in the state. All this is paralysed and completely stopped.

Attacked with courage and devotion already active in those directly associated with the library, and aided by such adequate appropriations as the legislature will be generally expected to make, in the present emergency, there may be quickly reared from and upon the ashes of the cherished old stores of books a greater state library, even more worthy of the wealth, supremacy, and status in the educational world, of the Empire state, and even more comprehensive and potentially useful, than it is likely that the old library would have become in the same time.

To such end and that it may be reached at the earliest possible day, the Regents of the University respectfully advise and earnestly request immediate, sufficient, sanctioning appropriations from the legislature. This request should be granted without any unnecessary delay.

